# Tuberculosis in international immigrants: Profile and vulnerability of cases residing in the municipality of São Paulo, Brazil

**DOI:** 10.1016/j.jmh.2022.100083

**Published:** 2022-01-28

**Authors:** Denise Gonçalves, Rubia Laine de Paula Andrade, Antônio Ruffino Netto

**Affiliations:** a Departamento de Medicina Social, Faculdade de Medicina de Ribeirão Preto, Universidade de São Paulo, Ribeirão Preto, SP, Brazil; b Departamento Materno-Infantil e Saúde Pública, Escola de Enfermagem de Ribeirão Preto, Universidade de São Paulo, Ribeirão Preto, SP, Brazil

**Keywords:** Epidemiology, Tuberculosis, Human migration, Emigrants and immigrants

## Abstract

**Objective:**

To analyze the profile of immigrants with tuberculosis (TB) and to identify the associated vulnerability characteristics.

**Methods:**

A cross-sectional study which used TB-WEB data from cases residing in São Paulo in 2016 (203 immigrants and 6,069 non-immigrants). The variables were analyzed using prevalence ratio and confidence intervals.

**Results:**

Among the immigrant cases, 67% were Bolivians. When compared to non-immigrants, immigrants were younger and frequently indigenous or presenting yellow ethnicity. They were also associated with a higher education level. We observed less immigrants having extrapulmonary TB and comorbidities, such as HIV/AIDS, diabetes mellitus, or drug use. Compared to cured cases, immigrants were not associated with treatment default and death, but they were associated with transfer to another state/country.

**Conclusions:**

Younger individuals and higher education levels were identified among immigrants, as well as a lower occurrence of comorbidities and drug use. It is believed that these results have led immigrants to more favorable outcomes of TB treatment.

## 1 Introduction

The scenario of migrations reflects a crisis in demographic regulation among countries, making space for a new migration modality, which inclusively involves Brazil. South-South migratory flow accounts for 36% of all world migratory flow (World Health Organization 2017), emerging because of many restrictions imposed in the South-North direction.

Movements in Latin America are examples of this concept, from the “Free Movement Agreement” of MERCOSUL (United Nations, Department of Economic and Social Affairs 2016), which allows the free circulation of citizens from countries belonging to the block.

The movement process and migrants’ new life conditions have a great influence over their health. Therefore, individuals infected by *Mycobacterium tuberculosis* may face determinants throughout their course or establishment in the destination country, which makes them vulnerable to developing the active infection of tuberculosis (TB) (Ruffino-Netto, 2002; Dhavan et al., 2017; Garbete and Antunes, 1997).

The immigrant population, especially those in an unregulated situation, are those who tend to take on the worst jobs (Fernandez and Ortega, 2008; Garcıa et al., 2009), have the worst housing conditions (López Salinas and Teixeira, 2020; Tasleem et al., 2020) and face more barriers to access health services due to language and not having a valid identification document (Garcıa et al., 2009). These barriers comprise a lack of information about how to use health services, financial problems, cultural differences, and others (Kalich et al., 2016).

In countries with low TB incidence, the groups of immigrants coming from other countries have higher disease incidence coefficients than the autochthonous population (Schneeberger et al., 2010; Heldal et al., 2008; Kik et al., 2011). However, this relationship has been poorly addressed in countries with high TB incidence (Pescarini et al., 2017).

As a result of the universalization of the Health Unified System (SUS) in Brazil, every immigrant whether being legal or not has the right to receive proper healthcare (Pescarini et al., 2017; Aguiar and Mota, 2014), which is extended to TB prevention and treatment.

The municipality of São Paulo (MSP) registered 385,120 immigrants in the National Registration System for Foreigners (SINCRE) of the Department of the Federal Police (DPF) in 2016, corresponding to 32% of all immigrants registered in Brazil and 3.2% of the city's population (São Paulo Cosmópolis, organizador 2017). The city is therefore identified as an attraction pole for this group of individuals, mainly because they look for new job opportunities.

A search for studies on immigrants with TB in Brazil was conducted in the LILACS, Scopus, and Pubmed databases and found seven manuscripts published in the period from 2008 to 2018.

Most studies in this search reported a predominance of Bolivians with TB (Aguiar and Mota, 2014; Martinez et al., 2012; Goldberg, 2013), in addition to analyzing the vulnerability of immigrants who face barriers against access to health services (Aguiar and Mota, 2014; Martinez et al., 2012; Steffens and Martins, 2016; Silveira et al., 2016) and precarious life and work conditions in their establishment in the country (Pescarini et al., 2017; Goldberg, 2013; Steffens and Martins, 2016) which has led them to develop the most active form of TB. All of the studies (Pescarini et al., 2017; Aguiar and Mota, 2014; Martinez et al., 2012; Goldberg, 2013; Steffens and Martins, 2016; Silveira et al., 2016; Pinto et al., 2018) were carried out in the MSP and three manuscripts (Pescarini et al., 2017; Goldberg, 2013; Pinto et al., 2018) addressed TB epidemiological aspects in immigrants.

Therefore, this study aims to analyze the profile and the characteristics of vulnerability associated with the disease-affected immigrants in the largest and most populous city in Brazil (São Paulo).

## 2 Methods

This is a cross-sectional study performed through secondary data collection. The study population consisted of the total number of TB cases in immigrants (203) and non-immigrants (6069), residing in MSP and notified on the TB-WEB database during the year 2016. Cases with diagnostic change as a treatment outcome were excluded from the study.

The data were obtained through the TB-WEB database and included the following - Variable of main interest: nationality; Exposure variables: Sociodemographic variables (age; gender; ethnicity; schooling); Clinical variables (case type; clinical form; associated diseases); Diagnostic and treatment variables (rapid molecular test; sputum smear microscopy; microscopy of other material; sputum culture; culture of other material; chest x-ray; other x-ray; anti-HIV and Directly Observed Treatment).

The Mann-Whitney test was used to test the difference in age between immigrants and non-immigrants, since the variable did not meet the assumption of homoscedasticity by Levene's test.

We calculated the prevalence ratio (PR) and the 95% confidence interval (95% CI) to identify the characteristics of vulnerability associated with the immigrants affected by TB. This calculation was also used to estimate the probability of immigrants to become sick and to default treatment, death and transfer to another state/country.

The study was approved by the Ethics Committee on Research of the Clinical Hospital of the Ribeirão Preto Medical School, in accordance with protocol no. 2.947.095.

## 3 Results

A total of 203 immigrants reported with TB in the MSP presented 24 different nationalities, with a greater (137 - 67%) concentration of Bolivians. This number is about ten times higher than Haitians, who accounted for the second highest number of cases (11 – 5.4%).

The other countries that immigrants with TB came from were: Peru (9 – 4.4%), China (8 – 3.9%), South Africa and Japan (5 – 2.5% each), Angola, Congo and Thailand (3 – 1.5% each), Germany, United States, Greece and Nigeria (2 – 1% each), Colombia, South Korea, Cuba, Ecuador, Spain, France, Ghana, Guinea-Bissau, Paraguay, Portugal and Dominican Republic (1 – 0.5% each).

The PR of 1.09 [95% CI - 0.94;1.25] indicated similar probabilities of sickness for both groups of immigrants and non-immigrants. According to this study, mean age of TB cases in immigrants was 32.8 (±15.7) years and 39.2 (±16.8) years in non-immigrants (*p*<0.0001).

Compared to non-immigrants, we found less immigrants among caucasian people (PR 0.47 [95% CI - 0.32;0.70]) and more among indigenous (PR 18.38 [95% CI – 13–37; 25.26]) and people who presented yellow ethnicity (PR 8.96 [95% CI – 5.86; 13.70]). Immigrants were also associated with a higher education level (8 to 11 years of education - PR 1.66 [95% CI – 1.10; 2.48] and 12 years or more - PR 2.16 [95% CI – 1.35; 3.48]) (Table 1).

**Table 1:** Distribution of tuberculosis cases between immigrants and non-immigrants living in the city of São Paulo and reported on TB-WEB, according to sociodemographic variables, 2016.

Variables		Immigrants*N* = 203n (%)	Non-immigrants*N* = 6069n (%)	PR (CI 95%)
**Sex**	Male	137 (67.4)	4.063 (66.9)	1
	Female	66 (33.5)	2.006 (33.0)	0.98(0.73–1.30)
Ethnicity*	Black	26 (13.7)	802 (14.3)	1.00(0.65–1.56)
	Caucasian	35 (18.4)	2.345 (41.8)	0.47(0.32–0.70)
	Indigenous	31 (16.3)	23 (0.4)	18.38(13.37–25.26)
	Brown	77 (40.5)	2.388 (42.6)	1
	Yellow	21 (11.1)	54 (1.0)	8.96(5.86–13.70)
Education*	None to 3 years	14(9.4)	683 (14.2)	0.94(0.51–1.75)
	4 to 7 years	34 (22.8)	1565 (32.6)	1
	8 to 11 years	69 (46.3)	1890 (39.4)	1.66(1.10–2.48)
	12 or more years	32 (21.5)	664 (13.8)	2.16(1.35–3.48)

⁎ Invalid responses (blank/ignored) of these variables were not considered for the analyses, therefore, the sample size in these analyzes did not correspond to the total population included in the study.

We observed less immigrants among patients with extrapulmonary TB (PR 0.59 [95% CI - 0.38–0.92]) and presenting associated diseases (PR 0.32 [95% CI - 0.25;0.42]), such as AIDS (PR 0.44 [95% CI - 0.24;0.81]), diabetes mellitus (PR 0.21 [95% CI - 0.07;0.66]), or drug use (PR 0.20 [95% CI - 0.10;0.40]) (Table 2).

**Table 2:** Distribution of tuberculosis cases between immigrants and non-immigrants living in the city of São Paulo and reported on TB-WEB, according to clinical variables, 2016.

Variables			Immigrants*N* = 203n (%)	Non-immigrants*N* = 6069n (%)	PR (CI 95%)
**Case type**	New case		180 (88.7)	5002 (82.4)	1
	Relapse		10 (4.9)	449 (7.4)	0.63(0.33–1.18)
	Retreatment		13 (6.4)	618 (10.2)	0.59(0.34–1.04)
**Clinical form**	Pulmonary		171 (84.2)	4773 (78.6)	1
	Extrapulmonary		21 (10.3)	1013 (16.7)	0.59(0.38–0.92)
	Pulmonary + Extrapulmonary		11 (5.4)	283 (4.7)	1.08(0.59–1.97)
**Associated diseases**	Yes		86 (42.3)	4274 (70.4)	0.32(0.25–0.42)
	No		117 (57.6)	1795 (29.5)	1
	AIDS	Yes	11 (5.4)	706 (11.6)	0.44(0.24–0.81)
		No	192 (94.5)	5363 (88.3)	1
	Diabetes Mellitus	Yes	3 (1.4)	412 (6.7)	0.21(0.07–0.66)
		No	200 (98.5)	5657 (93.2)	1
	Mental disorder	Yes	1 (0.4)	100 (1.6)	0.30(0.04–2.14)
		No	202 (99.5)	5969 (98.3)	1
	Drug use	Yes	8 (3.9)	1060 (17.4)	0.20(0.10–0.40)
		No	195 (96.0)	5009 (82.5)	1

Table 3 shows less immigrants among patients with positive result for microscopy of other material other than sputum (PR 0.20 [95% CI - 0.06;0.69]), and with a positive HIV-test result (PR 0.40 [95% CI - 0.22;0.74]) – Table 3.

**Table 3:** Distribution of tuberculosis cases between immigrants and non-immigrants living in the city of São Paulo and reported on TB-WEB, according to diagnostic and treatment variables, 2016.

Variables		Immigrants*N* = 203n (%)	Non-Immigrants*N* = 6069n (%)	PR (CI 95%)
Rapid Molecular Test*	Rifampicin-sensitive Mtb	80 (39.4)	1746 (28.7)	1.14(0.67–1.93)
	Rifampicin-resistant Mtb	1 (0.4)	60 (0.9)	0.43(0.06–3.16)
	Mtb with indeterminate resistance	0 (0.0)	13 (0.2)	0.90(0.06–14.30)
	Not detected Mtb	16 (7.8)	400 (6.5)	1
Sputum Smear Microscopy*	Positive	79 (38.9)	2478 (40.8)	0.85(0.61–1.17)
	Negative	65 (32.0)	1717 (25.8)	1
Microscopy of other material*	Positive	12 (5.9)	5050 (8.3)	0.20(0.06–0.69)
	Negative	3 (1.4)	245 (4.0)	1
Sputum Culture*	Positive	108 (53.2)	2318 (38.1)	1.13(0.76–1.69)
	Negative	29 (14.2)	709 (11.6)	1
Culture of other Material*	Positive	5 (2.4)	325 (5.3)	0.83(0.24–2.85)
	Negative	5 (2.4)	270 (2.4)	1
Chest X-ray*	Suspected tuberculosis	142 (69.9)	4193 (69.0)	1.52(0.75–3.07)
	Normal	8 (3.9)	363 (5.9)	1
	Other disease	5 (2.4)	104 (1.7)	2.13(0.71–6.37)
Other X-ray*	Suspected tuberculosis	4 (1.9)	118 (1.9)	0.23(0.03–1.79)
	Normal	1 (0.4)	6 (0.1)	1
	Other disease	1 (0.4)	38 (0.6)	0.18(0.01–2.55)
Anti-HIV*	Positive	11 (5.4)	751 (12.3)	0.40(0.22–0.74)
	Negative	170 (83.7)	4569 (75.2)	1
**Directly Observed Treatment**⁎⁎	No	50 (24.6)	1654 (27.2)	0.85(0.62–1.16)
	Yes	145 (71.4)	4041 (66.6)	1

⁎ Exams not performed or with invalid responses (blank/ignored) or results were not considered for the analyses, therefore, the sample size in these analyzes did not correspond to the total population included in the study.

⁎⁎ Invalid responses (blank/ignored) of these variables were not considered for the analyses, therefore, the sample size in these analyzes did not correspond to the total population included in the study. Mtb – Mycobacterium tuberculosis.

The cure rate among immigrants and non-immigrants was 68.4% and 70.9%, treatment default 17.2% and 16.7%, death 4.8% and 9.8%, transfer to another state/country 5.9% and 0.8%, and other outcomes 3.3% and 1.6%, respectively. In comparison with being cured, immigrants were not associated to treatment default (PR 1.05 [95% CI - 0.78–1.43]) or to death (PR 0.55 [95% CI - 0.30–1.00]), but they were associated with transfer to another state/country (PR 6.53 [95% CI – 3.56–11.96]).

## 4 Discussion

It is known that there is slowness between visa application and issue, thus, immigrants may face a situation of vulnerability to disease development, especially if they came from high burden TB countries and have already been infected by the bacillus (Heldal et al., 2008; Van der Werf and Lönnroth, 2014; Rennert-May et al., 2016; Tsang et al., 2017). Bolivia, Peru, Haiti, and China presented higher coefficients of TB incidence in 2017 than Brazil with 111, 166, 181 and 63 per 100 thousand inhabitants, respectively (World Health Organization 2018).

Despite this, the probability of sickness was similar in both groups of immigrants and non-immigrants, possibly due to the fact that Brazilians face social disparities which does not differ them from foreigners who come to the country to search for a job opportunity.

It is important to emphasize that this search happens worldwide and is made by a young population who migrates into neighboring countries without their parents or caregivers, but with the support of a migration network which charges high fees to formalize the migration or facilitates an illegal one, as well as access to local employment (Huijsmans, 2015).

In this study, TB affected immigrants and non-immigrants in a similar way when we consider their sex and we can see a larger proportion of males diagnosed with the disease in both evaluated groups.

This result arises the hypothesis that the social construction of gender increases the risk of developing the disease in males (Slama et al., 2007; Lönnroth et al., 2008), since they present more risk behaviors, such as underuse of health services, alcohol and drug consumption and high-exposure behaviors which can lead to infect them with HIV (Chikovore et al., 2020).

Bolivia is considered a multiethnic and multicultural country, where 74% of the population is indigenous and 15% *mestiza* (Silva, 2005). Considering that Bolivians accounted for 67% of TB cases in 2016, it is not a surprise to find more immigrants among indigenous ethnicity and yellow people and less immigrants with caucasian ethnicity.

The index of immigrants with higher education levels arriving to Brazil is about 26% (Kapa, 2018) higher than the autochthonous population. Therefore, it is entirely justifiable to identify more people who present a higher education level among immigrants.

However, immigrants are exposed to precarious work conditions which may arise from communication constraints due to different languages and lack of the documentation required to get a formal job (Sterud et al., 2018). Furthermore, we also have to mention the economic crisis that affected Brazil before the study period, increasing the unemployment rates and decreasing foreign investments in the country (Villen et al., 2017).

The study identified less immigrants among those who have extrapulmonary TB, as well as with positive-microscopy of materials other than sputum. It is known that extrapulmonary TB occurs with increased frequency in persons with underlying immunodeficiency caused by other health conditions such as HIV/AIDS and diabetes mellitus, which were more prevalent in Brazilian natives (Barreto-Duarte et al., 2020).

In turn, we verified less immigrants among those people with AIDS and a positive anti-HIV test. Brazil was considered by the World Health Organization (WHO) on the list countries with high burden of TB/HIV confection in 2016 (World Health Organization 2018), while Bolivia was not a country with high HIV incidence (Martinez et al., 2012; Joint United Nations Programme on HIV/AIDS (UNAIDS) 2018). Hypothetically, if there were a larger number of cases from African and Asian countries, the study results could be different from those which we found (Zammarchi et al., 2014).

Martinez et al. (Martinez et al., 2012) also found less immigrants with diabetes mellitus. Although studies report that patients with this disease have greater probability of becoming sick from TB (Beraldo et al., 2021; Demlow et al., 2015), such contrariness may be justified by the younger age of the immigrant population analyzed in this study and since an association between TB and diabetes is more frequently found in the age range between 50 and 69 years (Suwanpimolkul et al., 2014).

Although immigrants were not associated with mental disorders, a study shows some situations that affect their mental well-being: precarious immigration status, employment discrimination, social isolation, socioeconomic pressures, sociocultural stress, and lack of appropriate mental health supports (Alaazi et al., 2021). Such disorders include drug consumption, which was less frequent among immigrants when compared to the native people in this study and others (Paulino et al., 2016; Mor et al., 2013).

The higher frequency of immigrants with transference to another state/country is justified since they are constantly moving, even within the same city. However, treatment default is similar and proportionally high among immigrants and non-immigrants, showing the necessity of improving the access to Directly Observed Treatment or another strategy to monitor treatment compliance and moving homes.

We further have to mention the importance of “Immigrant Primary Care” in Sao Paulo created in order to address the barriers to immigrants to access the health services and which inclusively hire Bolivians to be its community health workers (Losco and Gemma, 2019).

Upon this reality, it is evident that the health system of MSP was capable of acting in detecting and treating immigrants affected by TB in the midst of several limitations such as communication constraints because of the language, perception of symptoms, and the stigma of the disease. It is important to highlight that the MSP had developed strategies to overcome these limitations and to provide measures for TB control in the Latin-American community (Steffens and Martins, 2016).

Although the authors recognize some limitations in this study, it may contribute to the production of knowledge concerning TB in immigrants. The limitations include: possible information bias given the use of secondary data, as they are likely of sub notification; many (393) notified cases in TB-WEB database were excluded from the study because the “nationality” was not fulfilled.

The main strength of the article is the approach of the migration in the largest city of Brazil. However, more studies are needed, especially those with a cohort design.

## 5 Conclusion

It was possible to identify that TB prevalence in non-immigrants does not seem to differ from the prevalence in immigrants; however, it is important to consider the possibility of having sub-notified cases. Among the identified immigrants, natives from Bolivia, indigenous and people with yellow ethnicity were the most frequent.

In addition, it was possible to identify younger individuals with higher education levels among immigrants, as well as lower occurrence of extrapulmonary TB and comorbidities such as AIDS, diabetes mellitus or drug use. We believe that these results led immigrants with TB to be less vulnerable to unfavorable treatment outcomes; moreover, it is a reflex of the strategies employed in the city in providing care to this population.

By virtue of these results, it seems that immigrants are more exposed to elements of social vulnerability than individual and programmatic vulnerability, as according to the literature, Bolivians are more exposed to precarious life and work conditions. Although there might be bias in the study, our research enables to construct hypotheses and lead the way to new research studies involving migration and TB in developing countries like Brazil.

## CRediT authorship contribution statement

**Denise Gonçalves:** Conceptualization, Data curation, Writing – original draft, Writing – review & editing. **Rubia Laine de Paula Andrade:** Conceptualization, Data curation, Writing – review & editing. **Antônio Ruffino Netto:** Data curation, Writing – review & editing.

## Declaration of Competing Interest

The authors declare that they have no known competing financial interests or personal relationships that could have appeared to influence the work reported in this paper.

## References

[bib0001] World Health Organization (2017).

[bib0002] United Nations, Department of Economic and Social Affairs (2016). http://www.un.org/en/development/desa/population/migration/data/estimates2/data/Number%20of%20international%20migrants.zip.

[bib0003] Ruffino-Netto A. (2002). Tuberculose: a calamidade negligenciada. Rev. Soc. Bras. Med. Trop..

[bib0004] Dhavan P., Dias H.M., Creswell J., Weil D. (2017). An overview of tuberculosis and migration. Int. J. Tuberc. Lung Dis..

[bib0005] Garbete M.J., Antunes M.L. (1997). Tuberculose em imigrantes. Saúde em números.

[bib0006] Fernandez C., Ortega C. (2008). Labor market assimilation of immigrants in Spain: employment at the expense of bad job-matches?. Span. Econ. Rev..

[bib0007] Garcıa A., Lopez-Jacob M., Agudelo-Suarez A., Ruiz-Frutos C., Ahonen E., Porthéf V. (2009). Condiciones de trabajo y salud en inmigrantes (proyecto ITSAL): entrevistas a informantes clave. Gaceta Sanitaria.

[bib0008] López Salinas A., Teixeira C. (2020). Settlement and housing experiences of recent Mexican immigrants in Vancouver suburbs. GeoJournal.

[bib0009] Tasleem Z., Na'eim Ajis M., Abidin N.A.Z. (2020). Examining the housing experiences in Malaysia: a qualitative research on Pakistani immigrant labours. Int. Migration & Integration.

[bib0010] Kalich A., Heinemann L., Ghahari S. (2016). A scoping review of immigrant experience of health care access barriers in Canada. J. Immigrant Minority Health.

[bib0011] Schneeberger S.G., Helbling P., Zellweger J.P., Altpeter E.S. (2010). Screening for tuberculosis in asylum seekers: comparison of chest radiography with an interview-based system. Int. J. Tuberc. Lung Dis..

[bib0012] Heldal E., Kuyvenhoven J.V., Wares F., Migliori G.B., Ditiu L., Fernandez de la Hoz K., Garcia D. (2008). Diagnosis and treatment of tuberculosis in undocumented migrants in low- or intermediate-incidence countries. Int. J. Tuberc. Lung Dis..

[bib0013] Kik S.V., Mensen M., Beltman M., Gijsberts M., Van Ameijden E.J.C., Cobelens F.G.J., Erkens C., Borgdorff M.W., Verver S. (2011). Risk of travelling to the country of origin for tuberculosis among immigrants living in a low-incidence country. Int. J. Tuberc. Lung Dis..

[bib0014] Pescarini J.M., Rodrigues L.C., Gomes M.G.M., Waldman E.A. (2017). Migration to middle-income countries and tuberculosis: global policies for global economies. Globalizat. Heal..

[bib0015] Aguiar M.E., Mota A. (2014). The family health program in the bom retiro district, São Paulo, Brazil: communication between Bolivians and healthcare workers. Interface (Botucatu).

[bib0016] São Paulo Cosmópolis, organizador (2017).

[bib0017] Martinez V.N., Komatsu N.K., Figueredo S.M., Waldman E.A. (2012). Equity in health: tuberculosis in the Bolivian immigrant community of São Paulo, Brazil. Trop. Med. Int. Health.

[bib0018] Goldberg A. (2013). Un abordaje comparativo en torno a la incidencia de la tuberculosis en inmigrantes bolivianos de Buenos Aires y São Paulo. REMHU Rev. Interdiscip. Mobil. Hum..

[bib0019] Steffens I., Martins J. (2016). Falta um Jorge": a saúde na política municipal para migrantes de São Paulo (SP). Lua. Nova.

[bib0020] Silveira C., Goldberg A., Silva T.B., Gomes M.H.A., Martin D. (2016). O lugar dos trabalhadores de saúde sobre os processos migratórios internacionais e saúde. Cad. Saúde Pública.

[bib0021] Pinto P.F.P.S., Neto F.C., Almeida Ribeiro M.C.S. (2018). Tuberculosis among South America immigrants in São Paulo municipality: an analysis in space and time. Int. J. Tuberc. Lung. Dis..

[bib0022] Van der Werf J.M., Lönnroth K. (2014). Pre-entry, post-entry, or no tuberculosis screening?. Lanc. Infect..

[bib0023] Rennert-May E., Hansen E., Zadeh T., Krinke V., Houston S., Cooper R. (2016). A step toward tuberculosis elimination in a low-incidence country: successful diagnosis and treatment of latent tuberculosis infection in a Refugee Clinic. Canad. Respirat. J..

[bib0024] Tsang C.A., Langer A.J., Navin T.R., Armstrong L.R. (2017). Tuberculosis among foreign-born persons diagnosed ≥10 years after arrival in the United States, 2010–2015. MMWR Morb. Mortal. Wkly. Rep..

[bib0025] World Health Organization (2018). http://www.who.int/tb/country/data/profiles/en/.

[bib0026] R. Huijsmans, Children and young people in migration: a relational approach. In: C.N. Laoire, A. White, T. Skelton, Movement, Mobilities and Journeys, Geographies of Children and Young People, vol 6, Springer, Singapore, 2015.

[bib0027] Slama K., Chiang C.Y., Enarson D.A., Hassmiller K., Fanning A., Gupta P., Ray C. (2007). Tobacco and tuberculosis: a qualitative systematic review and meta-analysis. Int. J. Tuberc. Lung Dis..

[bib0028] Lönnroth K., Williams B.G., Stadlin S., Jaramillo E., Dye C. (2008). Alcohol use as a risk factor for tuberculosis–a systematic review. BMC Publ. Heal..

[bib0029] Chikovore J., Pai M., Horton K.C., Daftary A., Kumwenda M.K., Hart G., Corbett E.L. (2020). Missing men with tuberculosis: the need to address structural influences and implement targeted and multidimensional interventions. BMJ Global Health..

[bib0030] Silva S.A. (2005).

[bib0031] R. Kapa, Unesco: qualificação de imigrantes no Brasil é melhor que a dos brasileiros. O Globo, 19 nov. 2018. https://oglobo.globo.com/sociedade/educacao/unesco-qualificacao-de-imigrantes-no-brasil-melhor-que-dos-brasileiros-23245990?fbclid=IwAR0Thq87kRHbuSoHc7PYkekcGcbvZJJlfIocj63MhEFUCS_BsX-CnTVKfWA.

[bib0032] Sterud T., Tynes T., Mehlum I.S., Veiersted K.B., Bergbom B., Airila A., Johansson B., Brendler-Lindqvist M., Hviid K., Flyvholm M.A. (2018). A systematic review of working conditions and occupational health among immigrants in Europe and Canada. BMC Publ. Heal..

[bib0033] Villen P. (2017). Immigrant labour in Brazil (2007-2015): the legal and the undocumented circuits. RUDN J. Econ..

[bib0034] Barreto-Duarte B., Sterling T.R., Fiske C.T., Almeida A., Nochowicz C.H., Smith R.M., Barnett L., Warren C., Blackman A., Lapa e silva J.R., Andrade B.B., Kalams S.A. (2020). Increased frequency of memory CD4+ T-cell responses in individuals with previously treated extrapulmonary tuberculosis. Front. Immunol..

[bib0035] World Health Organization (2018).

[bib0036] Joint United Nations Programme on HIV/AIDS (UNAIDS), Country: Bolivia, UNAIDS, Geneva, 2018. http://www.unaids.org/en/regionscountries/countries/bolivia.

[bib0037] Zammarchi L., Bartalesi F., Bartoloni A. (2014). Tuberculosis in tropical areas and immigrants. Mediterr. J. Hematol. Infect. Dis..

[bib0038] Beraldo A.A., Andrade R.L.P., Pinto E.S.G., Silva-Sobrinho R.A., Saita N.M., Monroe A.A., Villa T.C.S. (2021). Tuberculosis and diabetes mellitus: sociodemographic and clinical profile in Brazilian municipalities. Rev. Gaúcha Enferm..

[bib0039] Demlow S.E., Oh P., Barry P.M. (2015). Increased risk of tuberculosis among foreign-born persons with diabetes in California, 2010-2012. BMC Publ. Heal..

[bib0040] Suwanpimolkul G., Grinsdale J.A., Jarlsberg L.G., Higashi J., Osmond D.H., Hopewell P.C. (2014). Association between diabetes mellitus and tuberculosis in United States-born and foreign-born populations in San Francisco. PLoS ONE.

[bib0041] Alaazi D.A., Meheralli S., Diaz E., Hagadoren K., Punjani N., Salami B. (2021). Perspectives of service agencies on factors influencing immigrants’ mental health in alberta. Canada. IHTP.

[bib0042] Paulino J., Martins A., Machado M., Gomes M., Gaio A.R., Duarte R. (2016). Tuberculosis in native- and foreign-born populations in Portugal. Int. J. Tuberc. Lung Dis..

[bib0043] Mor Z., Kolb H., Lidji M., Migliori G.B., Leventhal A. (2013). Tuberculosis diagnostic delay and therapy outcomes of non-national migrants in Tel Aviv, 1998-2008. Euro. Surveill..

[bib0044] Losco L.N., Gemma S.F.B. (2019). Health subjects, territory agents: the community health agent in immigrant primary care. Interface (Botucatu).

